# Analysis of the Process and Results of High-Pressure Abrasive Water Jet Multilayer Cutting of Electrical Steel

**DOI:** 10.3390/ma17010094

**Published:** 2023-12-24

**Authors:** Monika Szada-Borzyszkowska, Wojciech Kacalak, Łukasz Bohdal, Wiesław Szada-Borzyszkowski

**Affiliations:** Department of Mechanical Engineering, Koszalin University of Technology, Racławicka 15-17 Street, 75-620 Koszalin, Poland; monika.szada-borzyszkowska@tu.koszalin.pl (M.S.-B.); lukasz.bohdal@tu.koszalin.pl (Ł.B.); wieslaw.szada-borzyszkowski@tu.koszalin.pl (W.S.-B.)

**Keywords:** high-pressure abrasive water jet cutting, electrical steel, cut surface, electrical steel single layers, bundle cutting, magnetic properties

## Abstract

Electrical steels are magnetically soft materials and are widely used in the electrical industry for the construction of power transformer cores, distribution transformers, current transformers, and voltage transformers. An important parameter of electrical components, which determines the efficiency of devices, is energy loss during remagnetization. Energy losses are caused by eddy currents, hysteresis, and magnetic delay associated with the low quality of the cut edge after the cutting of steels, and material deformations and excessive stress concentration in the surrounding cutting zones. Common techniques for cutting electrical materials in industrial lines include mechanical cutting and laser cutting. Work has shown that mechanical cutting of electrical steel single layers results in the occurrence of large deformation zones, and in cutting processes with a *high-pressure abrasive water jet (AWJ)*, significant uplifts of material and burrs at the bottom edges of sheets occur. The problem of increasing the cutting quality was solved through selecting the stream parameters for bundle cutting of electrical steels. It has been shown that in the process of cutting electrical sheet bundles, the height of burrs on the cut surface and the zone of plastic deformation are reduced. The work also presents comparison and analysis of characteristic features of the cut edge of electrical sheets obtained through *high-pressure abrasive water jet* and mechanical cutting processes. The influence of the type and processing parameters on the characteristic features of the material hysteresis loop was determined.

## 1. Introduction

Laminates for magnetic cores used in many electrical devices are made through mechanical cutting (punching, blanking, guillotining, and shear-slitting) or laser cutting. These processes interfere with the structure and properties of the material in the area of the cut edge, which negatively affects its magnetic properties [1]. Mechanical cutting or laser cutting of electrical sheets causes stresses, deformations, build-ups, and carbon deposits in the area of the cutting edge, which affect the magnetizing behavior and losses of the material core. In order to reduce the stress values in the area of the cut edge and improve the magnetic properties after cutting, it is recommended to stress-relieve the laminates. However, this increases production costs. Another option to obtain optimal magnetic properties of the material and a high-quality cut edge after cutting may be to select a cutting method that does not cause excessive changes in the area of the cut edge, which worsen the magnetic characteristics [2]. Analysis of the current state of knowledge shows that the cutting technologies used change the structure and properties of the material to varying degrees. Mechanical cutting such as blanking or shear-slitting requires precise control of many processing parameters depending on the thickness of the material being shaped. These are most often cutting speed [3], cutting clearance [4], rounding of the edges of cutting tools, rake angle of the knife (guillotining), or vertical clearance (shear-sitting) [5]. If there is no knowledge of how to select these parameters, the products obtained after the process may have numerous defects, such as roll-over and wavy cut edges, burrs, and edge cracks [6]. A very important problem in mechanical cutting processes is determining the influence of the cutting clearance, the shape of tool blades, tool wear mechanisms, and the force value, due to the quality of the obtained product. In the work [7], the influence of cutting clearance, friction conditions, sheet thickness, and punch size on the deformation of blanked sheet metal was examined. The influence of cutting clearance and friction coefficient on the size of the roll-over zone in the cut edge was examined numerically in [8], and the method of designing blanking tools was also presented. In the work [9], experimental studies were carried out on the influence of the size of the cutting clearance on the material cracking mechanisms, the built-up height, the length of the burnished zone, and the forces during aluminum blanking. The work [10] presents the optimization of the blanking process. The work [11] analyzed the influence of the clearance between the punch and the die on the lengths of individual zones on the cutting surface for AISI1045 material. The authors of the mentioned works showed that the clearance value should be determined depending on the set cutting speed and the type of material.

In the case of laser cutting, the main problem in the industry is the appropriate selection of the laser type and technological parameters of the cutting process in order to obtain a product of appropriate technological quality. Karnakis et al. [12] conducted experimental analyses of the laser cutting process of polymers and silicon nitride. Sotnikov et al. [13] analyzed the effect of the UV laser cutting process on the quality of the cut edge of silicon material. High cutting quality was achieved using a wavelength of 355 nm. The work [14] analyzed the influence of CO_2_ laser cutting process parameters such as current, nozzle work material gap, and cutting speed on the quality of the cut edge of composites. The authors demonstrated a significant effect of current and speed on the rate of material removal from the cutting zone. The work [15] analyzed the defects of products after processing of PLA/CNT nanocomposites in the form of thin plates, manufactured using MEX and cut with a CO_2_ laser. The aim of the work was to increase the accuracy of the shape of the products and reduce the roughness of the cutting surface. The most common defects of products created after laser cutting on production lines include: the occurrence of build-ups on the cutting surface, excessive heat affected zones, and a lack of complete separation of the material.

Currently, one of the unconventional methods of shaping materials is abrasive water jet processing. It can replace traditional techniques of processing electrical sheets. Shaping can take place in a wide range of materials, such as metals and their alloys [16], composites [17], glass [18], and marble [19]. The technology has numerous advantages. These include, among others, no thermal deformations in the cutting zone, high flexibility, high processing versatility, and high quality of the cutting surface [20]. It enables cutting materials of various hardnesses in a wide range of sheet thicknesses with low machining forces, without excessive interference in the cut surface and properties of the workpiece [21]. Thanks to this, the high-pressure abrasive water jet is a versatile technological tool with an increasingly wide range of processing and applications.

Abrasive water jet technology can be used to cut difficult-to-cut materials such as titanium alloys or glass. In [22], a cutting strategy was developed using an AWJ of titanium alloy Ti6Al4V. The aim of the work was to improve the efficiency of the process, shorten the cutting time, and develop some tips for processing other hard materials. The work [18] analyzed the influence of abrasive water jet processing parameters on the shaping of glass objects. It was determined how these parameters determine the roughness of the cutting surface and its texture.

In processing electrical steel sheets using cutting techniques, it was found that it is difficult to avoid the formation of burrs and build-ups on the cutting edges, which may cause short circuits between the sheets in the package and thus contribute to the increase in eddy current losses [23]. Knowledge about the processes of abrasive water jet cutting of magnetic materials is limited and the number of publications is limited. The authors mainly focus on developing a cutting method that minimizes the deformation zone of the cut edge, which negatively affects the magnetic properties. In the work [24], the waterjet cutting process was tested, indicating the presence of a deformed zone after the process in the cutting zone of non-oriented FeSi sheets with a width of 1.5 mm. The authors used sheet metal with a thickness of 0.20 mm and 0.35 mm to study the punching and waterjet cutting process. The course of the magnetization curve and energy losses and its components as a function of frequency were determined. Current research problems also concern ensuring process conditions enable the same quality of the cutting surface is obtained throughout the thickness of the material. In the case of cutting thin sheets such as electrical steels, increasing the erosive potential of the cutting stream through increasing the working pressure of water, with an inappropriate distance of the stream outlet from the processed material, and inappropriate feed speed may result in shape errors of the cut edge (larger width in the upper cutting zone and more beveled walls), excessive roughness of the cutting surface, damage to the electrical insulating coating of steel [25], and failure to achieve appropriate tribological properties of the surface [26]. According to the authors of the work [27], the problem of cutting using AWJ technology is the concentration of large surface irregularities in the initial area of the cutting surface, which significantly worsens the quality of products and limits their use. The authors of the study [28] showed that there are four characteristic zones on the cutting surface after the AWJ process. In the fourth zone, strong structural deformations often occur. The work optimized the process to reduce defects occurring in zone four.

Cutting magnetic materials with a high-pressure abrasive water jet can be a much more effective technology compared to mechanical cutting because it does not require the replacement of punches and dies, which are subject to gradual wear, as occurs in mechanical cutting using blanking [29]. Cutting with such a technology also eliminates the influence of temperature on the properties of the cut surface [30,31]. However, this process is not yet sufficiently understood. The main aim of this work is to analyze the effect of the high-pressure abrasive water jet cutting process on the characteristic features of the cut edge of electrical sheet metal. This work analyzed how the main process parameters affect the quality of the cut edge, and guidelines were developed to obtain high-quality cut edges that can be used on industrial lines. The research was carried out taking into account the main factors influencing the generation of waste on production lines, such as excessive burrs formed on the cut edge, the occurrence of a deformation zone of a significant width in relation to the thickness of the sheet, and defects in the electrical insulating coating (cracks, build-up, and defects). An additional aim of the work was to compare and analyze the obtained results with those obtained after mechanical cutting of electrical sheets using shear-slitting. Due to the disadvantages of the cutting processes of single sheets, it was decided to conduct research on the process and results of cutting bundles of electrical sheets.

## 2. Materials and Methods

### 2.1. Experimental Test Stands

#### 2.1.1. High-Pressure Abrasive Water Jet Cutting 

**Figure 1:**
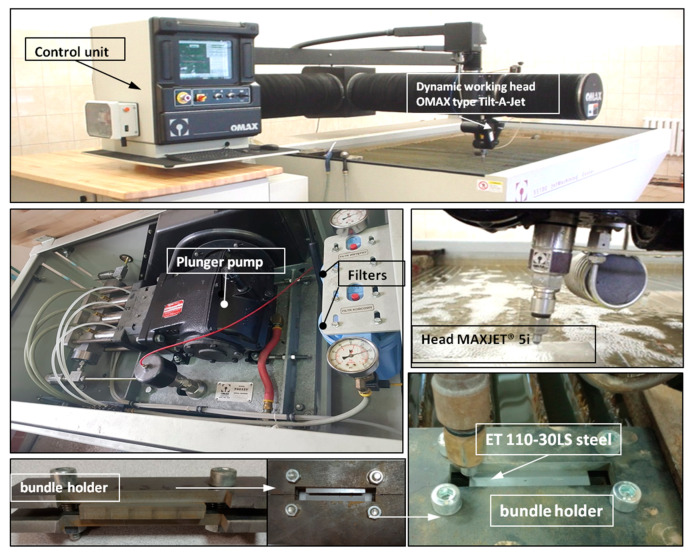
JetMachining Center 55100 machining center with Tilt-A-Jet ^TM^ head.

The high-pressure abrasive water jet cutting station consisted of a JetMachining Center 55100 machining center from the American company OMAX Corporation, Kent, WA, USA equipped with a Tilt-A-Jet^TM^ cutting head (Figure 1).

The Tilt-A-Jet ^TM^ head is equipped with a water nozzle with a diameter of 0.3556 mm and an OMAX MaxJet 5 mixing nozzle with a diameter of 0.762 mm. The abrasive material used when cutting AWJ was garnet [32,33] (almandine reigns as the favored garnet variety employed within AWJ technology) with granulation of 80 mesh. The distance between the nozzle and the workpiece was 1.65 mm. Single and bundled electrical sheets were cut at various feed speeds. The holder presented in Figure 1 was used for cutting bundles of electrical sheets.

#### 2.1.2. A Test Stand for Assessing the Geometric Structure of the Cut Surface and Shape Deviations of Individual Sheets and Their Bundles

**Figure 2:**
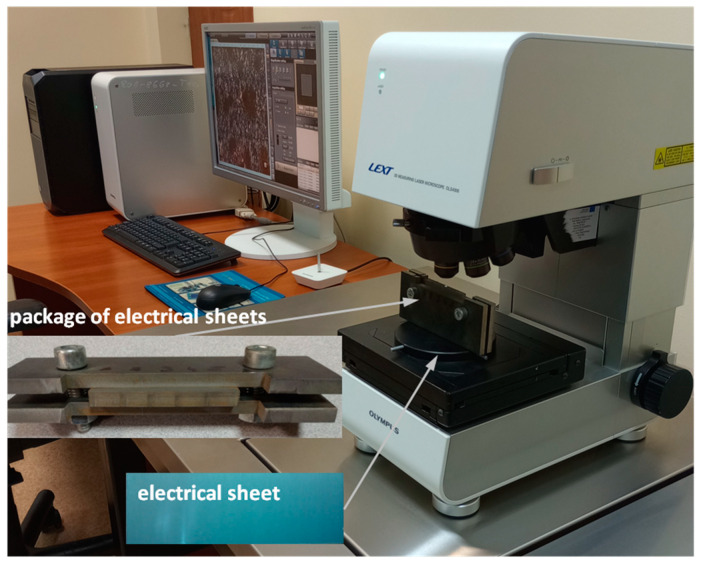
Station for measuring the geometric structure of the surface—LEXT OLS4000 laser confocal microscope from OLYMPUS.

The measurement of the geometric structure of the cut surface and shape deviation was performed using the LEXT OLS4000 laser confocal microscope from OLYMPUS (manufactured by Olympus, Tokyo, Japan), presented in Figure 2.

This microscope can obtain an image and perform accurate 3D measurements using the advanced optical system UIS2 Optical System—infinity correction, non-destructive method. Microscope parameters: total magnification 108×–17,280×, field of observation 2560 × 2560–16 × 16 µm, observation method BF/DIC/Laser/DIC confocal laser, 405 nm laser semiconductor laser, objectives 5×, 10×, 20×, 50×, and 100×, and magnification (zoom) optical 1×–8×.

To assess the edges of the electrical steel sheet after cutting with a high-pressure abrasive water jet, microscope settings were used, adopting a 10× objective and ×216 magnification. The measurement of a single layer of sheet metal was performed on the upper and side edges of the sample and from the front (Figure 2). Using the same microscope settings, the cut surface of a bundle of electrical sheets consisting of 20 sheets was analyzed after cutting (Figure 2). The measurement was also made on the lower and upper edges and from the front of the bundle. TalyMap Platinum (version 7.4) was used to analyze the results.

#### 2.1.3. A Stand for Testing the Mechanical Cutting Process

**Figure 3:**
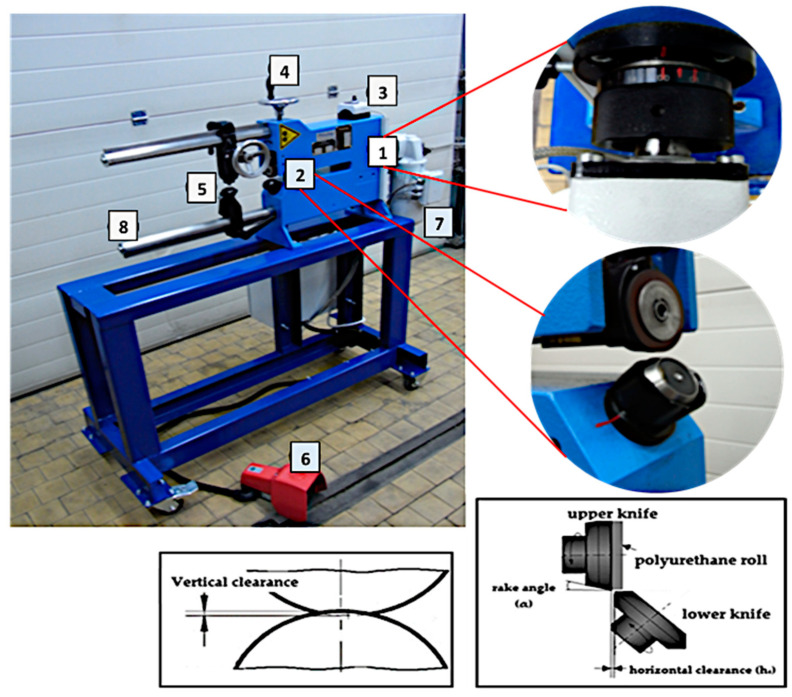
General view of shear-slitting device: 1—threaded socket with a scale for adjusting the clearance, 2—circular knives, 3—cutting speed regulator, 4—upper knife pressure regulator, 5—needle for attaching the sheet metal, 6—drive pedal, 7—engine, 8—scale for determining the diameter of the cut discs.

Mechanical cutting of the analyzed electrical steel was carried out using a station equipped with a shear-slitting device and the necessary auxiliary equipment (Figure 3). Using circular knifes, it is possible to cut strips of sheet metal and cut out disks or rings. This is possible because they are equipped with special holders and a needle. The machine is driven in single-stage, two-stage or stepless mode by an engine with a gear and a brake. The diameter of the cut discs and rings is determined using a scale. The sheet metal can be attached pneumatically or hydraulically. The clearance between the knives is adjusted via a threaded socket with a scale (Figure 3). 

### 2.2. Material Characteristics

ET 110-30LS electrical steel with a thickness of t = 0.3 mm was used in the tests. These are cold-rolled materials and belong to the grain-oriented group. After receiving the sheets from the supplier, they were tested for mechanical properties and hardness. They are used to build cores of power and distribution transformers, magnetic screens, and medium and high voltage transformers.

The sheets are covered on both sides with a thin inorganic coating with a thickness of 1.5–3.0 µm on the C-5 type side applied to a C-2 type substrate (labeled in accordance with ASTM A976-13: 2018 [34]). The basic mechanical properties of the material are summarized in Table 1. 

### 2.3. Conditions and Course of the AWJ Process and Mechanical Cutting 

The high-pressure abrasive water jet cutting parameters are detailed in Table 2. The mechanical cutting parameters are shown in Table 3.

Experimental tests of the cutting process using a high-pressure abrasive water jet were carried out at room temperature. The electrical steel sheet was placed in the working space of the JetMachining Center 55100 and protected against movement during the cutting process. Samples measuring 20 × 60 mm were cut from single sheet metal. The process was carried out for variable feed speeds. Three samples were cut for each setting. Then, a bundle of electrical steel consisting of 20 sheets was cut at various feed speeds. The samples were subjected to microscopic analysis on an OLYMPUS LEXT OLS4000 confocal laser microscope with a 10× objective and ×216 magnification. Edge profile measurements for a single electrical steel sheet were made in four places, and in each of these places 100 profiles were measured and the average was taken. Then, the cut surface of the sheet bundle was measured from the front and the upper and lower edges. In order to assess the height of the burr and the deformation zone, 3D maps were also developed.

The research on the shear-slitting process was carried out as follows: Strips 5 cm wide and 50 cm long were cut from sheet metal. The tests were carried out for variable values of cutting speed (v) and cutting clearance—horizontal clearance (h_c_)—in order to determine what settings ensure the highest quality of the cut edge. These are the main control parameters on production lines. Knives with a constant rake angle α = 7° and a constant vertical clearance c_v_ = 0.1 mm were used. After cutting, ET 110-30LS steel was examined in three planes, i.e., from the top, from the bottom, and from the front of the cutting surface, with measurements made for the same number of repetitions as for the cutting process with a high-pressure abrasive water jet, using a 10× objective and ×216 magnification.

## 3. Results

### 3.1. Experimental Research on the Process of Cutting Single Sheet Layers with a High-Pressure Abrasive Water Jet

**Figure 4:**
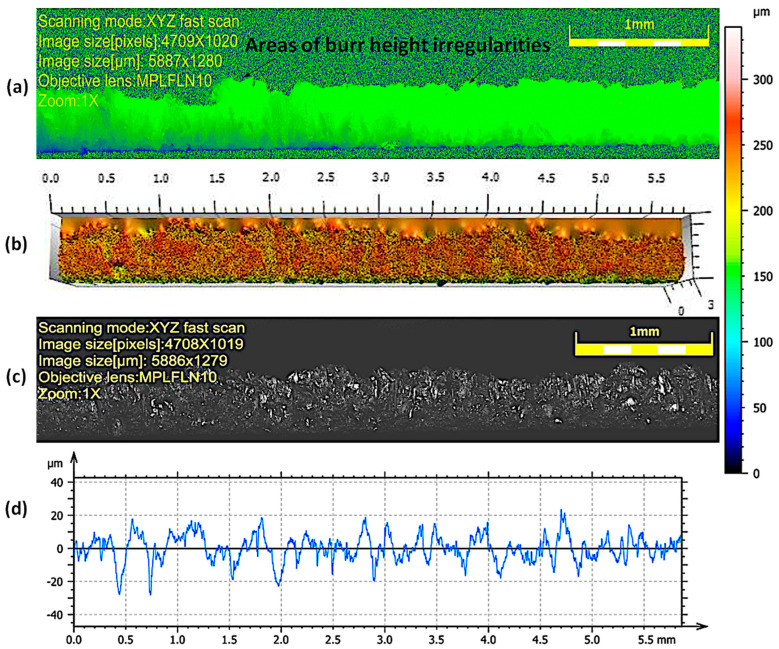
Appearance and profile of the cut surface of a sample cut at a speed of 1.29 m/min with a high-pressure abrasive water jet: (**a**) area of burr height irregularities, (**b**) 3D map, (**c**) enlarged photo of the surface—1× zoom, (**d**) roughness profile.

**Figure 5:**
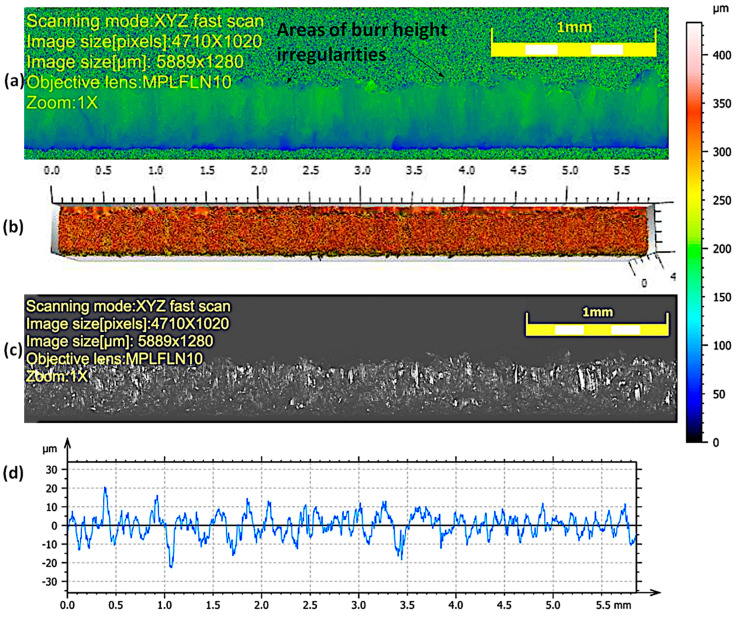
Appearance and profile of the cut surface of the sample with the lowest analyzed cutting speed (0.774 m/min) after cutting with a high-pressure abrasive water jet: (**a**) area of burr height irregularities, (**b**) 3D map, (**c**) enlarged photo of the surface—1× zoom, (**d**) roughness profile.

The view of the cut surface of selected samples after the high-pressure abrasive water jet cutting process is shown in Figure 4 and Figure 5. The cutting surface is characterized by slight waviness and granularity. Areas of impact of the abrasive are visible in the form of micro-grooves and grooves. For all samples, the occurrence of irregular micro-flows was observed, resulting from the accumulation of material on the lower edge of the sample during the cutting process. They may take the form of a burr. Burr formation has the effect of increasing the iron losses between the packaged steels in transformer cores and the damage of the insulation layers of sheets. The iron losses occur on the steel package in the form of heat depending on the short circuit lines formed between electrical steels [35].

Burrs are characterized by greater irregularity in height for high cutting speeds of 2.052–1.29 m/min. As the cutting speed decreases (0.972 and 0.774 m/min), the height and irregularity decreases (Table 4). This is due to the larger number of abrasive grains shaping the processed surface and the removal of excessive flashes on the lower edge of the sheet. The deviations in the roughness profiles measured for lower cutting speeds (0.972 and 0.774 m/min) have smaller deviations in values than those obtained for higher cutting speeds (2.052 m/min, 1.854 m/min, and 1.29 m/min), for which there are more values above 20 µm, which may cause local defects in the electrical insulating coating on the surface of the cut sheets.

**Figure 6:**
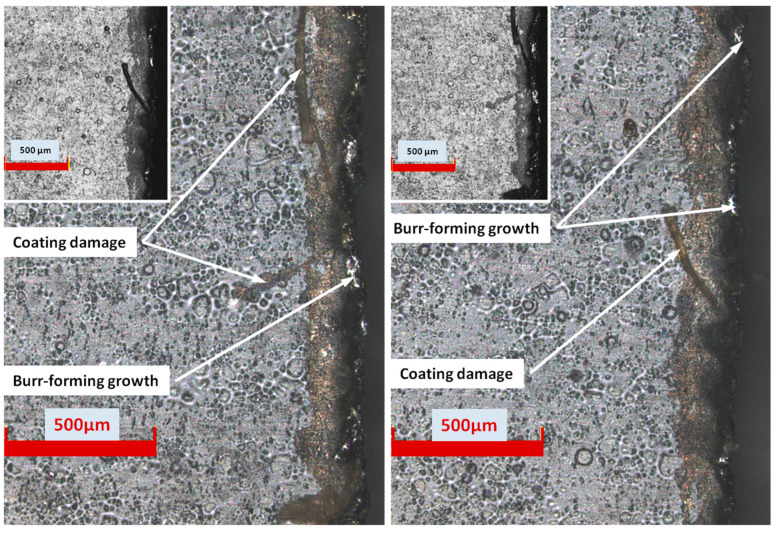
Appearance of the cut edge from the burr side with a visible deformation zone D_mz_ and damage to the electrical insulating coating (analyzed linear speed of cutting the material 1.29 m/min).

Figure 6 shows example photos of a bottom part of the sample of sheet metal after cutting, with the cutting edge and the visible area of deformation and local impact of the water-abrasive stream. After the cutting process of electrical steels, the magnetic properties of the product change along with the modification of the material texture in the deformation zone [36,37,38]. It is therefore necessary to minimize the width of this zone. Moreover, the authors of the works [25,35] indicate that damage to the electrical insulating coating in the vicinity of the cut edge may be caused by, among others, a wide zone of deformation after cutting. As the cutting speed decreased, the width of the deformed zone decreased. The minimum zone width was obtained, D_mz_ = 66 µm, for the highest analyzed cutting speed—0.774 m/min (Table 4). In the case of an average cutting speed of 1.29 m/min, this zone is characterized by non-uniformity with areas of material loss (Figure 6). Local areas of damaged coating in the form of cracks and grooves are also visible. There are also characteristic growths that also create local deformation zones. Local damage to the coating is probably caused by fragments of the abrasive water jet spreading from the bottom of the sheet.

Table 4 summarizes the obtained results and presents the values of the width of the deformation zone and the amount of burr obtained with specific parameters of the high-pressure abrasive water jet cutting process.

### 3.2. Experimental Research on the Process of Cutting Electrical Sheet Bundles with a High-Pressure Abrasive Water Jet

**Figure 7:**
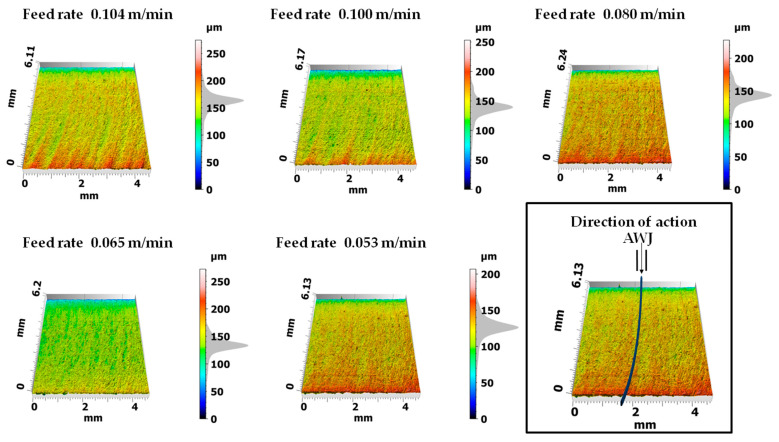
Surface topography of ET 110-30LS sheet metal bundles cut using an AWJ at different feed speeds.

Figure 7 presents 3D maps of the surface topography after cutting bundles of 20 layers of the analyzed sheet metal with a high-pressure abrasive water jet. The analyses of the surface topography show that the higher the cutting speed, the more visible are the machining traces in the form of curved grooves at the stream exit. However, for lower feed speeds of the cut sample, this trace disappears. The author of the work [35] carried out the multilayer punching process on electrical steel bundles composed of six sheets with a thickness of the entire stack of 2.1 mm. He pointed out problems related to selecting the appropriate cutting clearance, punches, and dies that would be able to withstand heavy loads. Since the production of the stator core requires quite accurate dimensions and tolerances of laminates, multilayer punching also requires extremely precise production of punches and dies and the design of appropriate process kinematics. As the obtained results indicate, slight wear of cutting tools leads to the formation of burrs and deformation zones as well as to the joining of sheet metal stacks. Cutting sheet metal packages using an AWJ with appropriate pressure in the stack ensures a reduction in the impact of plastic strains inside the material and the absence of conditions conducive to the formation of burrs and built-up edges. As a result, the quality of the cut edges of stacked sheets is significantly higher than in the case of multilayer punching (Figure 7).

**Figure 8:**
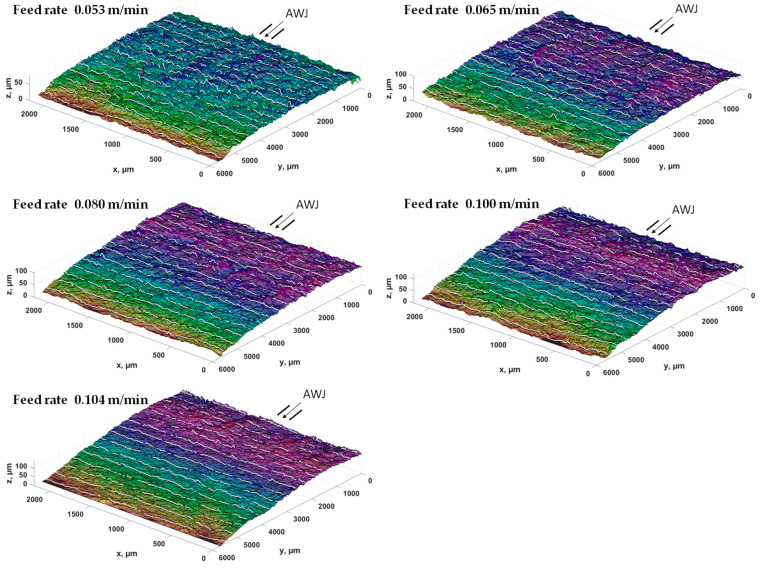
Convex profiles on the surfaces of electrical sheet bundles for different cutting speeds.

Figure 8 shows the surface profiling of sheet metal packages in the context of using different cutting speeds using a high-pressure abrasive water jet. The experiment was carried out taking into account the direction of entry of a high-pressure abrasive water jet along the y axis. The entry point of the jet was at level 0 and the exit point was at 6000 μm, as marked in Figure 8. The obtained convex profiles on the sheet metal surfaces present different features depending on the cutting speed. At lower speeds, smaller structural deformations and clearer cutting edges are observed. However, as the cutting speed increased, greater structural deformations were observed on the surface of the sheets, which resulted in less regular profiles (Figure 8, speeds 0.104 m/min and 0.100 m/min).

Processing in bundles using a high-pressure abrasive water jet is characterized by rounding of the upper edge in the highest layer of the stack, which is the result of the impact of the stream on initial contact with the treated surface. Table 5 presents the cutting parameters for sheet metal bundles. The table indicates the numbers of sheets selected from the stack, where number 20 means one layer from the bottom of the bundle. 

The analysis of the obtained values of the burr height and the width of the deformation zone showed that the lowest (last) layer on the lower edge has much smaller burrs than in the cutting process of single layers (Table 4 and Table 5) cut with a high-pressure abrasive water jet and also in the mechanical cutting process. The remaining middle layer of the package (18 layers) has burrs not exceeding h_z18_ < 30 µm (average value of 18 layers) at the highest cutting speed of 0.104 m/min and h_z18_ < 20 µm at the lowest cutting speed. The values of the width of the deformation zone are similar, with a smaller effect observed for the middle layers of the bundle, D_mz18_ = 74 µm at the highest cutting speed of 0.104 m/min and D_mz18_ = 40 µm for the lowest cutting speed of 0.053 m/min.

### 3.3. Experimental Research on the Mechanical Cutting Process

**Figure 9:**
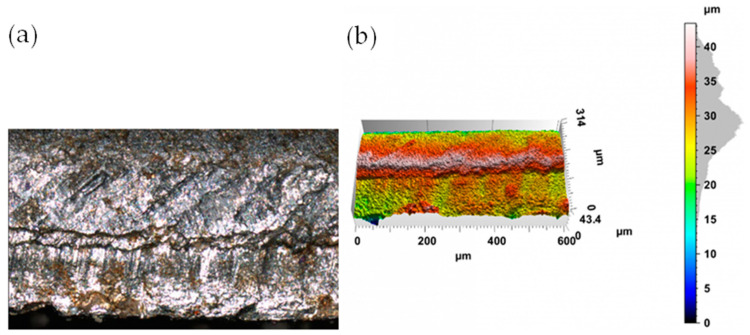
The view of the surface of the cut edge (h_c_ = 0.04 mm and v = 10.2 m/min): (**a**) surface photography, (**b**) surface topography.

**Figure 10:**
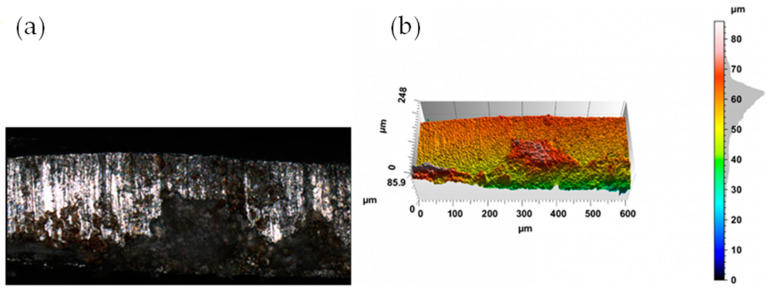
The view of the surface of the cut edge (h_c_ = 0.08 mm and v = 10.2 m/min): (**a**) surface photography, (**b**) surface topography.

The appearance of the cut surface of selected samples after the mechanical cutting process is shown in Figure 9 and Figure 10.

In the case of mechanical cutting processes, after the cutting process, fracture irregularities appear on the cutting surface, which depend primarily on the cutting clearance h_c_. Samples cut with a clearance of hc = 0.04 mm and a cutting speed of v = 10.2 m/min are characterized by a clear transition from the slip to dissolution fracture (Figure 9). A slip fracture occurs as a result of the plastic flow phase and the displacement of the material fibers relative to each other. When the material cracks, a crack zone with a matte appearance is created. The fracture zone is often characterized by high roughness and frequent shape deviations caused by asymmetric cracks occurring during the separation process, which, with excessive cutting clearance, may also result in the formation of tears on the cut edge. Increased roughness of the cutting surface is often related to the presence of a crack zone in the entire cross-section of the material. Obtaining a sliding break across the entire thickness of the sheet metal is practically impossible when cutting with shear- slitting. This is possible in adiabatic blanking. Increasing the cutting clearance resulted in an increase in the width of the slip fracture zone. In the case of cutting with values h_c_ = 0.08 mm and speed v = 10.2 m/min, the samples were characterized by a matt slip fracture (Figure 10). Increasing the clearance causes an increase in the build-up and an expansion of the concentration of plastic deformations. The coating also curves towards the center of the sheet metal cross-section.

In mechanical cutting processes, burrs occur as a result of a significant extension of the duration of the plastic flow phase, which causes a delay in the cracking phase when the clearance between the tools is too large and the speed is too high. Conditions are then created that cause cracks to run at an angle to the sheet surface and not in a straight line, which favors the formation of not only built-up edges and burrs but also edge bends. Through using the appropriate cutting clearance h_c_, it is possible to obtain a cut edge without rounding and burr areas, but this is difficult to achieve in the case of electrical steel sheets. Based on the conducted research, it was found that the process of mechanical cutting with shear-slitting can obtain a minimum width of the deformed zone at the level of D_mz_ = 113 µm, which is more than obtained during high-pressure abrasive water jet cutting at the highest speeds, both when cutting a single sheet of metal (D_mz_ = 91 µm) and a bundle of sheets (D_mz_ = 84 µm). This means that for each speed setting during the AWJ cutting process, it is possible to obtain a smaller width of the deformed zone, which may reduce the negative effect of the machining process on the magnetic properties. Obtaining the smallest width of the deformation zone in the mechanical cutting process requires the use of very small cutting clearances, h_c_ = 0.02 mm, which may be difficult to precisely set on production lines, and an increase in the cutting clearance will result in an increase in the deformation zone. Cutting with clearance h_c_ = 0.1 mm and cutting speeds above v = 30 m/min is particularly unfavorable because the deformation zone, due to the increase in momentum in the cutting zone and the increased flow area, reaches a value of up to D_mz_ = 198 µm. In the mechanical cutting process, using an appropriate cutting clearance h_c_, it is possible to obtain a cut edge without rounding and burr zones, but this is difficult to achieve in the case of electrical sheets. The minimum burr height was obtained for a cutting speed of v = 20 m/min and cutting clearance of h_c_ = 0.03 mm. The minimum burr height was h_z_ = 71 µm.

### 3.4. Magnetic Characteristics 

**Figure 11:**
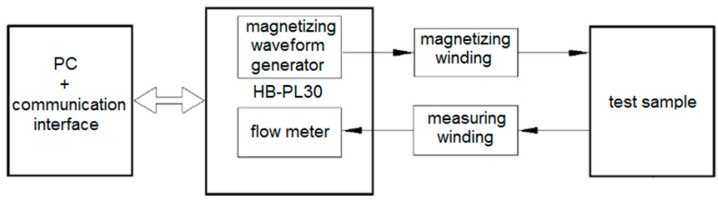
Diagram of the test stand for testing magnetic characteristics after mechanical and *high-pressure abrasive water jet* cutting.

An important scientific aspect is to determine the extent to which the tested conditions of the high-pressure water-abrasive cutting process and mechanical cutting affect the magnetic properties. Specially prepared samples made of electrical steel ET 110-30LS were used for the tests (samples with a closed ring, external diameter D = 120 mm, and internal diameter d = 90 mm). Sample preparation was performed in accordance with the standards and procedures presented in [26]. Measurements of the magnetic characteristics were made for the determined values of the amplitude of the magnetic field strength H_m_ = 250 A/m. The frequency of the demagnetizing waveform was 10 Hz. The tests were carried out on a test stand consisting of the components shown in Figure 11.

**Figure 12:**
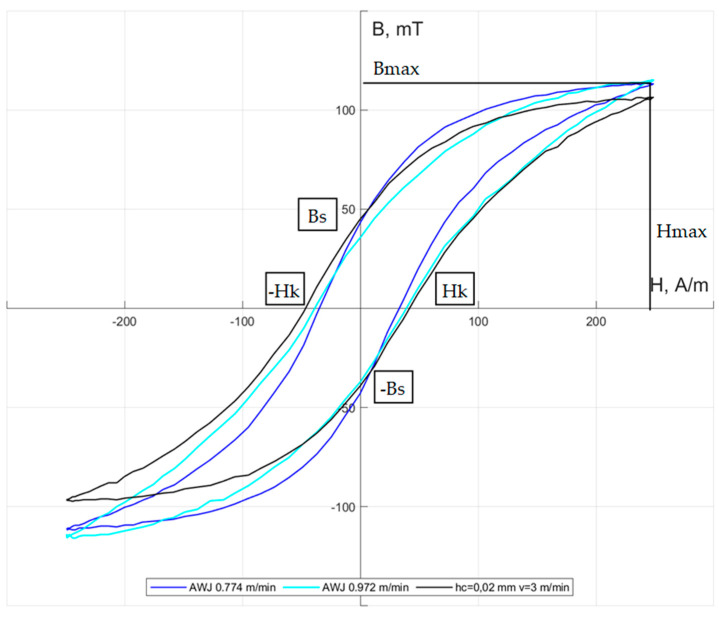
The influence of the *high-pressure abrasive water jet* cutting process and mechanical cutting on the hysteresis loop of the tested electrical steel.

Figure 12 shows the influence of cutting technology on the hysteresis loops of ET 110-30LS steel.

As a result of plastic deformations, internal stresses, and an increase in temperature in the cutting zone, the hysteresis loops of electrical steels may undergo significant changes, resulting in an increase in their internal surface area and total losses. Based on the test results, it can be concluded that the distortions of the hysteresis loop are not excessive, but the influence of individual processes and parameter values on its characteristic features can be noticed. The intensity of H_max_ and the induction of B_max_ are called saturation intensity and induction, respectively. The value of B_s_ is called the remanence induction. The strength of the magnetic field H_k_ is called the coercive force. The hysteresis loop of a soft magnetic material is narrow due to small H_k_ values. The greatest changes in the shapes of the hysteresis loop occur in the areas of the upper bend of the characteristic and saturation. For the isolated mechanical cutting parameters and the highest analyzed AWJ cutting speed, the area of concentration of maximum plastic strains occurred only in the vicinity of the cutting edge of the material. Consequently, changes in the hysteresis loop mainly concern the characteristics of the saturation area and the maximum induction value. When the cutting speed increases, the saturation induction value decreases. Probably, the increase in the height of burrs and the width of the deformed zone results in an increase in the intensity of coercivity and the induction of remanence. For the lowest cutting speeds (v = 3–5 m/min), the highest maximum induction and the lowest coercivity occur. The mechanical cutting process carried out for selected parameters ensured similar changes in the characteristics of the hysteresis loop. The greatest differences in relation to *high-pressure abrasive water jet* cutting occurred in the area of coercivity (there was an increase in coercivity in relation to AWJ cutting). For mechanical cutting, there was an increase in the remanence induction compared to the lowest cutting speed with *high-pressure abrasive water jet* cutting.

## 4. Conclusions

In the case of cutting electrical steels using the *high-pressure abrasive water jet* cutting process, current knowledge in the literature is limited. This is a technology that is used to shape many materials, but in each case, it requires precise determination of process parameters and conditions so as to obtain products of appropriate technological quality, free from defects. Therefore, experimental analyses were carried out to determine the possibility of shaping electrical steels using a high-pressure abrasive water jet. The influence of process conditions on selected features of the cut edge of ET 110-30LS steel was analyzed. The obtained results were compared with those obtained using traditional mechanical cutting. The benefits of multilayer cutting of electrical sheets have been demonstrated in the form of reduction of the deformed zone and burr height compared to mechanical cutting and cutting of single sheets using the AWJ method.

The influence of the tested cutting technologies on selected magnetic characteristics of the material was determined. The following conclusions emerge from the conducted research:

- AWJ technology can economically cut complex-shaped details from electrical sheets through selecting key processing parameters. It’s suitable for cutting tiny dimensions.
- Different cutting speeds in high-pressure abrasive water jet technology allow complete material separation through adjusting the clearance between cutting tools and cutting speed in the mechanical cutting process.
- Both AWJ and mechanical cutting processes create a zone subject to local deformation, which may adversely affect the micromechanical, microstructural, and magnetic properties of the workpiece near the cutting edge.
- The width of the cutting zone in high-pressure abrasive water jet cutting increases with speed. In mechanical cutting, it widens with both speed and clearance increments.
- AWJ significantly reduces the deformed zone compared to mechanical cutting, with reductions of about 42% at the lowest speed and 20% at the highest speed for single sheets. For sheet bundles, reductions reach approximately 54% at the lowest speed (64% for middle sheets) and around 25% (34% for middle sheets) at the highest speeds.
- When cutting sheets or bundles, AWJ creates uneven burrs on the sheet’s bottom, with the smallest height (29 µm) occurring in bundle cutting at the lowest speed.
- Across all 18 middle layers of the bundle (AWJ), the average burr height was less than 30 µm. In mechanical cutting, maintaining a minimum clearance value of 0.02 mm helps in reducing burr height.
- The *high-pressure abrasive water jet* cutting process causes the concentration of plastic deformations near the edge of the cutting surface and a smaller deformation zone than the mechanical cutting process, which reduces negative changes in the coercivity intensity and remanence induction. For mechanical cutting, when optimal processing parameters were used, there was an increase in remanence induction compared to the highest AWJ cutting speed.

## Figures and Tables

**Table 1:** Mechanical properties of coated ET 110-30LS steel (at *T* = 20 °C).

Rm [MPa]	R_p0.2_ [MPa]	Density [kg/m^3^]	F_m_ [kN]	A_g_ [%]	Hardness [HB]	Hardness [HV]
337	314	7800	0.96	10.94	157	164

**Table 2:** AWJ process parameters.

Parameters	Values
Water jet pressure, MPa	366.8
Abrasive mass flow rate, kg/min	0.256
Water jet orifice diameter, mm	0.38
Focusing tube diameter, mm	0.76
Standoff distance, mm	1.65

**Table 3:** Mechanical cutting process parameters.

Parameters	Values
Slitting velocity v, m/min	3−32
Horizontal clearance h_c_, mm	0.02−0.1
Rake angle α, ◦	7
Vertical clearance c_v_, mm	0.1

**Table 4:** Parameters of the cutting process of single sheet of electrical steel with a high-pressure abrasive water jet with the obtained width of deformation-affected zone and burr height.

lp	Pressure p, MPa	Feed Rate, m/min	Burr Height h_z_, µm	Deformation-Affected Zone D_mz_, µm
1	366	0.774	62	66
2		0.972	91	71
3		1.290	102	82
4		1.854	115	88
5		2.052	128	91

**Table 5:** Parameters of the cutting process of bundles composed of 20 layers of electrical steel sheets with a high-pressure abrasive water jet with the obtained width of the deformation-affected zone and burr height.

lp	Pressure p, MPa	Feed Rate, m/min	Height of a Burr h_z20_, µm	Deformation Zone D_mz20_, µm	Height of a burr h_z18_, µm	Deformation Zone D_mz18_, µm
1	366	0.053	29	52	19.2	40
2		0.065	49	62	24.2	44
3		0.080	64	56	26.7	52
4		0.100	67	82	27.4	60
5		0.104	72	84	28.2	74

## Data Availability

Data are contained within the article.
